# Potential interstitial lung abnormalities on chest X-rays prior to symptoms of idiopathic pulmonary fibrosis

**DOI:** 10.1186/s12890-022-02122-8

**Published:** 2022-08-30

**Authors:** T. W. Hoffman, H. W. van Es, D. H. Biesma, J. C. Grutters

**Affiliations:** 1 Interstitial Lung Diseases Center of Excellence, Department of Pulmonology, St. Antonius Hospital, Koekoekslaan 1, 3435 CM Nieuwegein, The Netherlands; 2 Department of Radiology, St. Antonius Hospital, Nieuwegein/Utrecht, The Netherlands; 3 Department of Internal Medicine, Leiden University Medical Center, Leiden, The Netherlands; 4 Division of Heart and Lungs, University Medical Center, Utrecht, The Netherlands

**Keywords:** Idiopathic pulmonary fibrosis, Interstitial lung abnormalities, Chest X-ray, Diagnostic delay, Survival

## Abstract

**Background:**

Idiopathic pulmonary fibrosis (IPF) often has significant diagnostic delay. At present it is not well-known what factors associate with time to diagnosis and if this is associated with survival after the diagnosis. There has also been increasing attention for interstitial lung abnormalities on chest CT-scans. In this study we assessed what factors associate with time to diagnosis in patients with IPF, and whether early stages of pulmonary fibrosis can be seen on chest X-rays prior to the start of symptoms.

**Methods:**

In this retrospective study, 409 Dutch patients with IPF were included. Clinical characteristics, including patient demographics, medical history, time of start of symptoms, time of first visit to pulmonologist, and any previous radiographic imaging reports were collected from patient records.

**Results:**

In 96 patients (23%) a chest X-ray was available that had been made prior to the start of symptoms (median of 50.5 months (IQR 26.3–83.3 months)), and this showed potential interstitial lung abnormalities in 56 patients (58%). The median time from the start of symptoms to the final diagnosis was 24.0 months (interquartile range 9.0–48.0 months). In a multivariate model that corrected for diffusion capacity of the lung for carbon monoxide, forced vital capacity, sex, and age at diagnosis, time to diagnosis did not associate with survival (hazard ratio 1.051 (95% CI 0.800–1.380; *p* = 0.72)).

**Conclusions:**

There is a significant diagnostic delay for patients with IPF, but longer time to diagnosis did not associate with survival. Interstitial lung abnormalities were seen in more than half of the patients in whom a chest X-ray had been made prior to the start of symptoms. This illustrates that a computed tomography scan should be strongly considered for analysis of unexplained abnormalities on a chest X-ray. This could facilitate early detection and possibly prevention of disease progression for patients with pulmonary fibrosis.

**Supplementary Information:**

The online version contains supplementary material available at 10.1186/s12890-022-02122-8.

## Background

Idiopathic pulmonary fibrosis (IPF) is a severe fibrotic interstitial lung disease (ILD) with a median survival of 3–5 years after diagnosis. No curative treatment is available except for lung transplantation, but the antifibrotic agents pirfenidone and nintedanib have been shown to slow disease progression. Therefore, timely diagnosis and/or referral to an Interstitial Lung Diseases centre is important [1]

Previous studies have shown that diagnostic delay for patients with IPF is common, and that consultations with a pulmonologist, as well as pulmonary function testing, chest CT-scans, and additional diagnostics are often performed in the years leading up to the eventual diagnosis [2]. Over half of all patients report having received another diagnosis prior to the diagnosis of IPF [3], and the final diagnosis is oftentimes made more than one year after the onset of symptoms [3–8]. Factors that lead to an increased time to diagnosis in patients with IPF include underreporting of ILD features on diagnostic testing and prolonged time to pulmonology referral from a primary care setting [9], as well as male sex, older age, and comorbid conditions such as cardiac and gastro-oesophageal disorders [4, 5].

In a study that was performed prior to the availability of antifibrotic agents, longer diagnostic delay was associated with worse survival, independent of disease severity at diagnosis [7]. However, in a more recent study, diagnostic delay, stratified by symptom onset > 1 year or ≤ 1 year prior to diagnosis, did not associate with survival [4]. In another study, patients who were referred to an ILD centre within 12 months of symptom onset had significantly longer survival compared to patients referred later. Yet, when corrected for disease severity at diagnosis, diagnostic delay no longer associated with survival in that study either [8].

Recently, there has been increased attention for interstitial lung abnormalities on chest computed tomography (CT) scans, as these likely represent an early stage of pulmonary fibrosis [10]. However, interstitial lung abnormalities are not defined on chest X-rays and it is not known if early stages of pulmonary fibrosis can be seen on chest X-rays of patients with IPF prior to the start of symptoms.

In the present study we investigated the patient trajectory prior to diagnosis in a large Dutch cohort of patients with IPF, including radiographic imaging that was performed prior to the start of symptoms, as well as factors associated with time to diagnosis and the relation between time to diagnosis and mortality.

## Methods

This was a retrospective study. All patients with a diagnosis of IPF between January 2011 and October 2017 that were included in the Biobank for ILD of St. Antonius Hospital were included. Participants of the Biobank have given permission for the use of their clinical data in scientific research (approved by the local ethics committee (MEC-U) under study number R05-08A). The study was performed in accordance with the declaration of Helsinki.

The diagnosis of IPF was always made by a multidisciplinary team, and this was done in accordance with the Fleischner Society recommendations and ATS/ERS/JRS/ALAT guidelines [11, 12]. Diagnoses were classified as either a consensus or a working diagnosis of IPF. A consensus diagnosis of IPF can be made when, in the appropriate clinical context of IPF, a definite pattern of usual interstitial pneumonia (UIP) is seen on the chest CT-scan, or after integration of clinical, radiographic and histopathologic findings during multidisciplinary discussion. All biopsy results included were from surgical lung biopsies. A working diagnosis of IPF can be made when a patient has a progressive fibrosis interstitial pneumonia in the absence of an alternative explanation, and IPF is thought to be the most likely diagnosis by the multidisciplinary team.

Clinical characteristics were retrieved from patient records. This included patient demographics, medical history, time of start of symptoms, time of first visit to pulmonologist, and, when available, any previous radiographic imaging reports. When the imaging report mentioned reticular abnormalities, interstitial abnormalities, infiltrative abnormalities, or pulmonary fibrosis, this was considered to represent potential interstitial lung abnormalities.

Patient follow up was completed up to December 2018. Study data were collected and managed using REDCap electronic data capture tools hosted at St. Antonius Hospital [13]. Data analyses were performed using IBM SPSS Statistics for Windows, version 26 (IBM Corp., Armonk, N.Y., USA). Median values with interquartile range (IQR) are reported for non-normally distributed continuous variables. For comparing two groups, Student’s t-test, Mann–Whitney U-test, Kruskall-Wallis test, Fisher’s exact test, or chi-squared test were used where appropriate. For survival analyses, Kaplan–Meier curves were made, and Log-rank tests and Cox-regression analyses were performed where appropriate. Patients were censored when lost to follow up, when they underwent lung transplantation, or at the time of data collection. A *p*-value < 0.05 was considered to represent statistical significance.

## Results

Four-hundred-and-nine patients were included. Baseline characteristics are provided in Table 1. Three-hundred-and-thirteen patients were male (77%), the median age at diagnosis was 68.2 years (interquartile range 62.0–74.5), and 78 patients (19%) were under 60 years of age at the time of diagnosis. Presenting complaints included cough in 284 patients (69%) and dyspnoea in 343 patients (84%). Nine patients (2%), did not have any respiratory complaints, of whom five (1%) were screened for pulmonary fibrosis because they had a family member with pulmonary fibrosis.

**Table 1:** Baseline characteristics for 409 patients with idiopathic pulmonary fibrosis

Total patients	409
Male (%)	313 (77)
Median age at diagnosis (IQR)	68.2 (62.0–74.5)
First-degree family member with pulmonary fibrosis (%)	116 (28)
Other family member with pulmonary fibrosis (%)	24 (6)
Current smoker (%)	33 (8)
Former smoker (%)	298 (73)
Never smoker (%)	78 (19)
Significant exposure to asbestos, dusts, fumes, or radiation (%)	79 (9)
Gastro-oesophageal reflux (%)	149 (36)
Cardiovascular disease (%)	188 (46)
Initial symptoms	
Cough	284 (69)
Dyspnoea	343 (84)
No respiratory complaints	9 (2)
Evaluation in context of screening of family members of patients with familial pulmonary fibrosis	5 (1)
Chest X-ray done prior to start of symptoms (%)	96 (23)
Showing potential interstitial lung abnormalities (%)	56 (58)
CT-imaging done prior to start of symptoms (%)	44 (11)
Showing interstitial lung abnormalities or pulmonary fibrosis (%)	34 (77)
Symptoms started directly after infectious episode (%)	40 (10)
Symptoms started directly after surgery (%)	5 (1)
Treatment with steroids prior to diagnosis (%) #	103 (25)
Treatment with other immunosuppressant prior to diagnosis (%)	23 (6)
Other ILD diagnosis prior to diagnosis of IPF (%)	67 (16)
Cardiology workup for symptoms prior to pulmonology visit (%)	45 (11)
Time from start of symptoms to first visit with pulmonologist, median months (IQR)	5.0 (2.0–12.0)
Time from first visit with pulmonologist to start of symptoms, median months (IQR)	10.0 (4.0–33.0)
Time from start of symptoms to diagnosis, median months (IQR)	24.0 (9.0–48.0)
Forced vital capacity at diagnosis, median percentage of predicted (IQR)	78.1 (64.0–91.3)
Diffusion capacity for carbon monoxide at diagnosis, median percentage of predicted (IQR)	41.0 (32.0–51.0)
Radiographic pattern at diagnosis	
UIP pattern (%)	238 (58)
Probable UIP (%)	101 (25)
Inconsistent with UIP or indeterminate for UIP (%)	70 (17)
Histology pattern at diagnosis	99 (24)
UIP pattern (%) *	70 (70)
Probable UIP (%) *	16 (16)
Inconsistent with UIP or indeterminate for UIP (%) *	13 (13)
Consensus diagnosis of IPF (%)	190 (47)
Received treatment with antifibrotic therapy after diagnosis (%)	331 (81)

^#^Prednisolone equivalent >  = 10 mg/daily for > 4 weeks

*Percentages represent the percentage of the patients in whom lung biopsy specimens were available

**Fig. 1:**
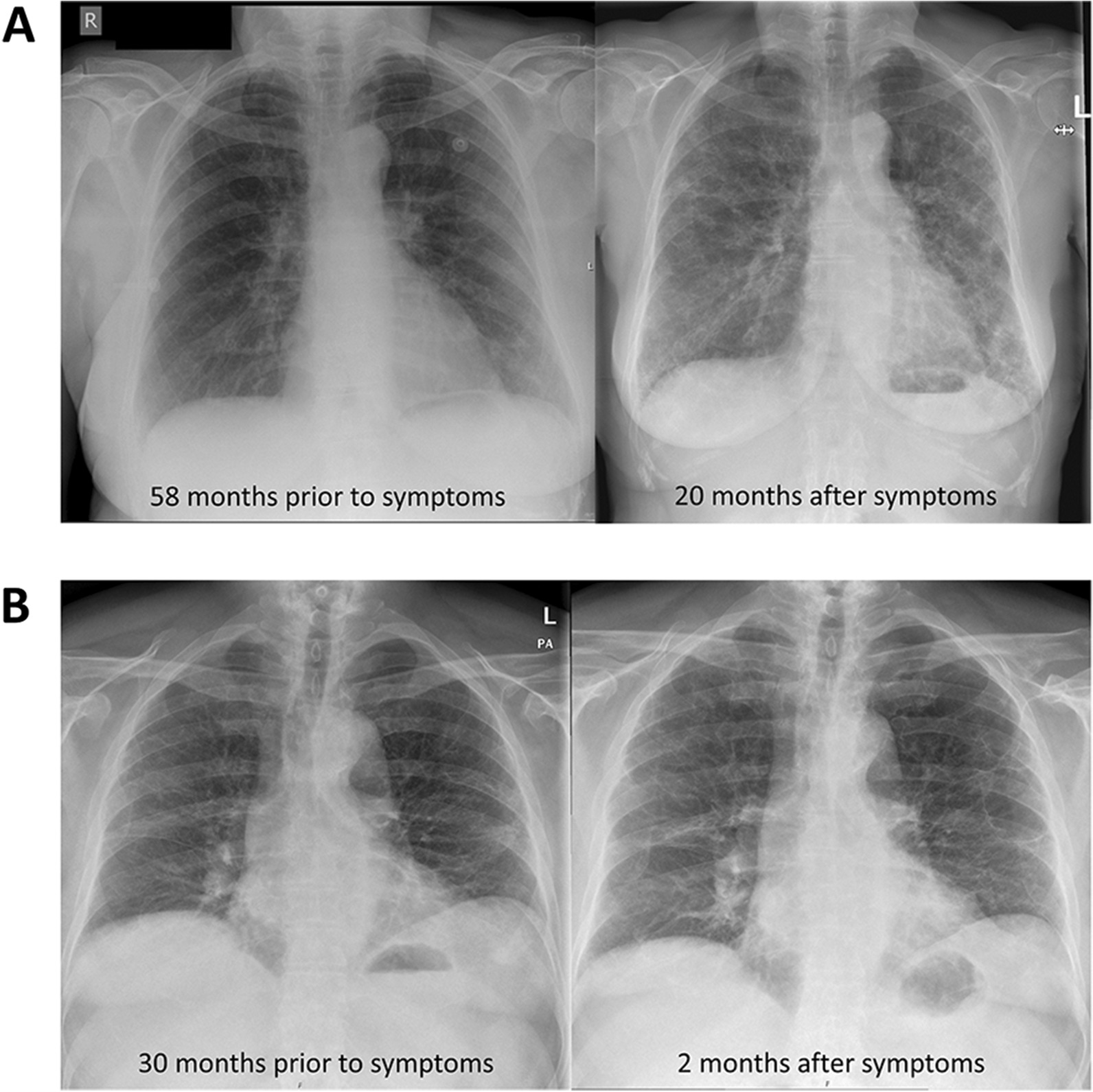
Examples of patients who had potential interstitial lung abnormalities on chest X-rays that were made prior to the start of symptoms. **A** patient who presented with temporary dyspnoea after a wasp sting 58 months prior to developing a cough and exertional dyspnoea. The chest X-ray at the time showed minimal reticular changes in the left lower lung. The chest X-ray that was made 20 months after symptoms started shows bilateral reticular changes in the lower fields, especially on the left. **B** patient who was evaluated for an episode of chest pain 30 months prior to developing a cough. The chest X-ray at the time showed bilateral reticular abnormalities. The chest X-ray that was made 2 months after symptoms started showed progressive reticular changes mainly in the lower fields

In 98 patients (23%) a chest X-ray was available that had been made prior to the start of symptoms, and this showed potential interstitial lung abnormalities in 56 patients (58%). Two examples are provided in Fig. 1. The indications for the chest X-rays are shown in Additional file 1: Table S1. Chest X-ray findings are described in more detail in Additional file 1: Table S2. The median time between the chest X-ray and the start of symptoms was 50.5 months (IQR 26.3–83.3 months). In 91 patients, the time between the chest X-ray and the start of symptoms was one year or more. The time between the chest X-ray and the start of symptoms did not significantly differ between patients in whom the chest X-ray did or did not show potential interstitial lung abnormalities (median 44.5 months (IQR 24.0–73.0 months) compared to 58.0 months (IQR 32.3–97.5 months); *p*-value 0.16). CT-imaging that had been done prior to the start of symptoms was available in 44 patients (11%), and showed interstitial lung abnormalities or pulmonary fibrosis in 34 patients (77%). Details on CT-imaging findings are provided in Additional file 1: Tables S2 and S3. In 23 of those 34 patients with abnormalities seen on CT-imaging a chest X-ray had also been made, and this showed potential interstitial lung abnormalities in seventeen patients (74%). A CT-scan, done prior to the start of symptoms, was available for 19 patients with potential interstitial lung abnormalities on chest X-ray, and showed abnormalities consistent with pulmonary fibrosis or interstitial lung abnormalities in 17 (89%).

Symptoms had started directly after a respiratory tract infection in 40 patients (10%) and directly after surgery in five patients (1%). The median time from the start of symptoms to the first visit with a pulmonologist was 5.0 months (IQR 2.0–12.0 months), and 45 patients (11%) had undergone evaluation by a cardiologist prior to their visit to a pulmonologist. The median time from the first visit with a pulmonologist to the final diagnosis was 10 months (IQR 4.0–33.0) and the median time from the start of symptoms to the final diagnosis was 24.0 months (IQR 9.0–48.0). In 209 patients (51%), the time from start of symptoms to the final diagnosis was less than 24 months.

The association between clinical characteristics and time to diagnosis is shown in Table 2. Factors that associated with longer time from the start of symptoms to diagnosis included having any family member with pulmonary fibrosis (median time to diagnosis 19.0 months (IQR 6.0–42.5 months) compared to 24.5 months (IQR 10.3–49.0); *p* = 0.03), no chest X-ray having been made prior to the start of symptoms (median time to diagnosis 17.0 months (IQR 7.0–34.5), compared to 26.0 months (IQR 10.0–50.8); *p*-value 0.01), symptoms that did not start directly after a respiratory tract infection (median time to diagnosis 25.0 months (IQR 10.0–49.0), compared to 9.5 months (IQR 5.0–27.8); *p* < 0.001), having received treatment with prednisolone prior to diagnosis (median time to diagnosis 37.0 months (IQR 19.0–78.0), compared to 18.5 months (IQR 8.0–41.3); *p* < 0.001), having received treatment with other immunosuppressive medication prior to diagnosis (median time to diagnosis 48.0 months (IQR 26.0–84.0), compared to 23.0 months (IQR 9.0–45.5); *p* = 0.002), and having received another ILD diagnosis prior to the diagnosis of IPF (median time to diagnosis 47.0 months (IQR 26.0–83.0), compared to 19.0 months (IQR 8.0–42.0); *p* < 0.001). Patients who had received another ILD diagnosis prior to the diagnosis of IPF had received treatment with prednisolone or other immunosuppressive medication significantly more often (69% versus 17%, *p* < 0.001; 15% versus 4%, *p* = 0.001).

**Table 2:** Clinical characteristics associated with time to diagnosis in 409 patients with IPF

	Time from symptom onset to diagnosis, months (IQR)		*p*-value
	Factor present	Factor absent	
Male sex (n = 313)	24.0 (9.0–47.5)	24.5 (8.3–49.8)	0.70
Age at diagnosis < 60 years (n = 78)	17.0 (6.8–45.0)	25.0 (10.0–49.0)	0.10
First-degree family member with pulmonary fibrosis (n = 116)	19.5 (6.3–41.8)	24.0 (10.0–49.0)	0.05
Other family member with pulmonary fibrosis (n = 24)	11.5 (5.3–57.8)	24.0 (9.0–48.0)	0.12
Any family member with pulmonary fibrosis (n = 125)	19.0 (6.0–42.5)	24.5 (10.3–49.0)	0.03
Current smoker (n = 33)	19.0 (5.5–39.0)		0.07
Former smoker (n = 298)	26.0 (10.0–52.0)		
Never smoker (n = 78)	23.0 (7.8–37.8)		
Significant exposure to asbestos, dusts, fumes, or radiation (n = 79)	26.0 (10.0–49.0)	23.0 (8.0–47.0)	0.18
Gastro-oesophageal reflux (n = 149)	24.0 (9.5–57.0)	24.0 (9.0–46.5)	0.49
Cardiovascular disease (n = 188)	24.5 (8.0–52.0)	24.0 (9.0–44.0)	0.35
Chest X-ray done prior to start of symptoms (n = 96)	17.0 (7.0–34.5)	26.0 (10.0–50.8)	0.01
Chest X-ray showing potential interstitial lung abnormalities (n = 56) *	17.0 (7.0–27.8)	21.0 (7.0–45.0)	0.34
Symptoms started directly after infectious episode (n = 40)	9.5 (5.0–27.8)	25.0 (10.0–49.0)	< 0.001
Symptoms started directly after surgery (n = 5)	12.0 (7.5–88.5)	24.0 (9.0–48.0)	0.99
Treatment with steroids prior to diagnosis (n = 103) #	37.0 (19.0–78.0)	18.5 (8.0–41.3)	< 0.001
Treatment with other immunosuppressant prior to diagnosis (n = 23)	48.0 (26.0–84.0)	23.0 (9.0–45.5)	0.002
Other ILD diagnosis prior to diagnosis of IPF (n = 67)	47.0 (26.0–83.0)	19.0 (8.0–42.0)	< 0.001
Cardiology workup for symptoms prior to pulmonology visit (n = 45)	23.0 (8.0–60.5)	24.0 (9.0–48.0)	0.59
Consensus diagnosis of IPF (n = 190)	23.0 (9.0–49.0)	24.0 (9.0–47.0)	0.75
Received treatment with antifibrotic therapy after diagnosis	25.0 (10.0–49.0)	19.5 (7.0–42.0)	0.17

*Compared to patients in whom a chest X-ray was performed but did not reveal potential interstitial lung abnormalities. # prednisolone equivalent >  = 10 mg/daily for > 4 weeks

**Fig. 2:**
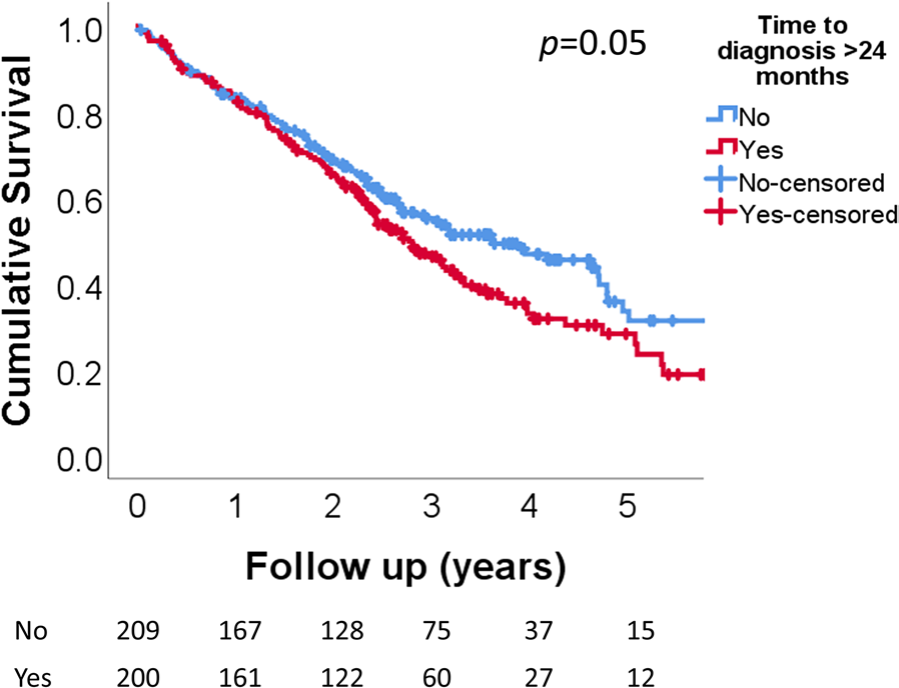
survival after diagnosis in 409 patients with IPF, stratified by time to diagnosis > 24 months or ≤ 24 months. median survival after diagnosis was 1012 days in patients with time to diagnosis > 24 months (interquartile range (IQR) 530–1863), compared to 1322 days in patients with time to diagnosis ≤ 24 months (IQR 610–2476) (*p* = 0.05). In a multivariate model that corrected for diffusion capacity of the lung for carbon monoxide, forced vital capacity, sex, and age at diagnosis, time to diagnosis did not associate with survival (hazard ratio 1.051 (95% confidence interval 0.800–1.380; *p* = 0.72)). At risk tables represent the number of patients at the start of every year during follow up

Time to diagnosis did not significantly associate with survival after diagnosis. In patients with time to diagnosis > 24 months, median survival after diagnosis was 1012 days (interquartile range (IQR) 530–1863), compared to 1322 days (IQR 610–2476) in patients with time to diagnosis ≤ 24 months (Fig. 2; *p* = 0.05). In a multivariate model that corrected for diffusion capacity of the lung for carbon monoxide, forced vital capacity, sex, and age at diagnosis, time to diagnosis did not associate with survival (hazard ratio 1.051 (95% CI 0.800–1.380; *p* = 0.72)). When using time to diagnosis as a continuous variable in the same multivariate model, this did not associate with survival either (hazard ratio 1.000 (95% CI 0.997–1.004; *p* = 0.97)). Factors that associated with survival in the same multivariate model included being a current smoker (hazard ratio 0.46 (95% CI 0.21–0.97); *p* = 0.04), a history of cardiovascular disease (hazard ratio 1.41 (95% CI 1.07–1.86); *p* = 0.01), and treatment with antifibrotic therapy (hazard ratio 0.52 (95% CI 0.37–0.75; *p* < 0.001) (Table 3).

**Table 3:** Factors associated with survival

	Hazard ratio (95% confidence interval)	*p*-value
First-degree family member with pulmonary fibrosis (n = 116)	1.18 (0.86–1.63)	0.30
Other family member with pulmonary fibrosis (n = 24)	1.35 (0.69–2.63)	0.38
Any family member with pulmonary fibrosis (n = 125)	1.18 (0.86–1.62)	0.29
Current smoker (n = 33) *	0.46 (0.21–0.97)	0.04
Former smoker (n = 298) *	1.01 (0.68–1.50)	0.97
Significant exposure to asbestos, dusts, fumes, or radiation (n = 79)	1.12 (0.85–1.47)	0.44
Gastro-oesophageal reflux (n = 149)	0.98 (0.75–1.30)	0.91
Cardiovascular disease (n = 188)	1.41 (1.07–1.86)	0.01
Chest X-ray prior to start of symptoms showing potential interstitial lung abnormalities (n = 56) #	1.65 (0.88–3.08)	0.56
CT-scan prior to start of symptoms showing potential interstitial lung abnormalities (N = 34) $	0.98 (0.37–2.58)	0.97
Symptoms started directly after infectious episode (n = 40)	0.82 (0.51–1.34)	0.43
Symptoms started directly after surgery (n = 5)	0.65 (0.16–2.64)	0.55
Treatment with steroids prior to diagnosis (n = 103) ^	1.10 (0.81–1.49)	0.55
Treatment with other immunosuppressant prior to diagnosis (n = 23)	0.94 (0.53–1.65)	0.82
Other ILD diagnosis prior to diagnosis of IPF (n = 67)	1.01 (0.71–1.44)	0.96
Cardiology workup for symptoms prior to pulmonology visit (n = 45)	0.96 (0.64–1.44)	0.83
Time from start of symptoms to first visit with pulmonologist (n = 409)	1.00 (0.99–1.01)	0.81
Time from start of symptoms to diagnosis (n = 409)	1.00 (1.00–1.00)	0.97
Consensus diagnosis of IPF (n = 190)	0.89 (0.68–1.17)	0.40
Received treatment with antifibrotic therapy after diagnosis	0.52 (0.37–0.75)	< 0.001

Hazard ratios are derived from a multivariate model that corrected for diffusion capacity of the lung for carbon monoxide, forced vital capacity, sex, and age at diagnosis. * compared to never smokers as a reference category. # compared to patients in whom a chest X-ray was performed but did not reveal potential interstitial lung abnormalities. $ compared to patients in whom a CT-scan was performed but did not reveal potential interstitial lung abnormalities. ^ prednisolone equivalent >  = 10 mg/daily for > 4 weeks

## Discussion

In this cohort of 409 patients with IPF from the Netherlands, a long time between the start of symptoms and the final diagnosis was common. The median time between the start of symptoms and the final diagnosis was 24 months, and the time to diagnosis was longer than 24 months in 49% of the patients. Most patients had an insidious onset of the disease, but in 10% of patients the first symptoms became apparent after a respiratory tract infection, and in 1% of patients after surgery. The insidious disease onset is also evidenced by the fact that in 96 patients a chest X-ray had been done for other reasons prior to the start of symptoms, and that 58% of those chest X-rays showed potential interstitial lung abnormalities. In the 44 patients for whom CT-imaging of the lungs was done prior to the start of symptoms, interstitial lung abnormalities were seen on 77% of the scans. The median time between the first visit to a pulmonologist and the final diagnosis was 10 months, indicating a significant doctor’s delay in addition to a patient’s delay in arriving at the final diagnosis.

Factors that associated with longer time to diagnosis were having no family member with pulmonary fibrosis, treatment with immunosuppressive medication prior to the diagnosis, having received another ILD diagnosis prior to the diagnosis of IPF, and symptoms not having started directly after a respiratory tract infection. In contrast to previous studies [4], we did not find an association between a history of cardiovascular disease or gastro-oesophageal reflux and longer time to diagnosis, nor was longer time to diagnosis associated with a cardiology consultation prior to the first visit to a pulmonologist.

Somewhat counterintuitively, longer time to diagnosis was not significantly related to shorter survival after diagnosis, also in a multivariate model that corrected for sex, age and lung function at diagnosis. This is in contrast to a previous study that was done prior to the availability of antifibrotic therapy [7]. However, this is in line with the findings from two more recent studies. [4, 8] Other than a clinical history of cardiovascular disease and being a current smoker, no other clinical factors associated with survival in our study. The absence of an association between longer time to diagnosis and survival is sobering, as this illustrates that despite our best efforts, we still have little to offer to IPF patients in order to prevent disease progression and prolong survival. In this cohort, it needs to be taken into account that approximately 80% of patients was treated with antifibrotic medication after they were diagnosed with IPF. Notably, pirfenidone and nintedanib do seem improve survival in patients with IPF according to meta-analyses of randomized controlled trials, but the actual magnitude of this effect is uncertain [14]. In our cohort treatment with antifibrotic therapy was also associated with better survival in a multivariate model, but we cannot exclude that this is due to selection bias.

The fact that a significant proportion of patients in whom either a chest X-ray or a CT-scan had been done for some other reason, prior to the start of symptoms related to IPF, is of interest. Interstitial lung abnormalities are found on CT-scans of 2–9% of older adults. [10] When followed up, interstitial lung abnormalities are oftentimes progressive, and they associate with respiratory symptoms and increased mortality [10]. However, as far as we are aware, it is not known what proportion of patients with IPF had potential interstitial lung abnormalities on previous imaging. The proportion of 58% that we found on chest X-rays is very high. Furthermore, these chest X-rays were done a median of 44.5 months prior to the start of symptoms, which indicates that subclinical disease can be present for a long time in many patients. This would indicate that screening of certain populations at high risk for IPF might be worthwhile, as there would be ample time for intervention an halting disease progression [15]. However, it is important to acknowledge that it is presently unknown whether treatment with antifibrotic medication can prevent progression of interstitial lung abnormalities [15]. Furthermore, it is not known whether the abnormalities seen on the chest X-rays represent the same clinical entity as interstitial lung abnormalities seen on chest CT-scans. The latter are often subtle changes that are not visible on chest X-rays, and might represent a much earlier stage of fibrosis.

We were not able to explore precisely how the radiology reports that mentioned potential interstitial lung abnormalities were interpreted by the requesting physician at the time that the imaging was done. In any case, the absence of (persisting) respiratory symptoms might have caused the physician at that time to refrain from further investigations or follow-up. Unfortunately, in these cases, this has meant that a rather easy opportunity for earlier diagnosis of pulmonary fibrosis or interstitial lung abnormalities was missed. A potential solution to be considered is that the radiologist report specifically advises to perform a chest CT-scan in case of potential interstitial lung abnormalities on a chest X-ray. Considering that 23% of this cohort had a chest X-ray done and 58% of chest X-rays showed abnormalities in this cohort, this would facilitate earlier diagnosis in at least one in eight patients with IPF. However, this might cause unnecessary CT-scans to be performed, and it is not known if this would lead to more timely diagnosis or recognition of patients with interstitial lung abnormalities that are a precursor to IPF.

Despite our findings, we do not think that chest X-ray is a useful screening tool for patients with suspected IPF, such as first-degree relatives of patients with pulmonary fibrosis [16, 17]. This is partially because chest X-ray is a cruder diagnostic tool, and some subtle abnormalities may be missed. Furthermore, in the context of recent studies in relatives of patients with pulmonary fibrosis, as well as lung cancer screening programs, more information will be available on the prognostic significance of interstitial lung abnormalities, as seen on CT-scan, than abnormalities seen on chest X-ray [10, 16, 17].

The present study has several limitations. First, this was a retrospective study, and this can always lead to missing data or incorrect categorization. Second, because patients estimated the time since the start of symptoms at the time of the first visit to a pulmonologist, this could be subject to recall bias, and may not be a completely reliable value. We are however confident that in most if not all cases, the imaging was done before the start of symptoms. In the majority of patients, the time between the chest X-ray and the reported symptom onset was one year or more, and for the patients in whom we were able to assess the reason for requesting the chest X-ray, this was not related to symptoms of IPF. Third, because radiographic imaging was oftentimes performed at another hospital, we are not certain that we were able to assess all chest X-rays and CT-scans that were done prior to the start of symptoms in our patients. In addition, we were not able to view the original images, and had to determine whether potential interstitial lung abnormalities were present based on the radiologist’s report. This could have led to underreporting of interstitial lung abnormalities.

## Conclusions

In this cohort of 409 Dutch patients with IPF, the median time between the start of symptoms and the final diagnosis was 24 months, with half of the patients having a time to diagnosis of over two years. In 23% of the patients, a chest X-ray that was made a median of 50.5 months prior to the start of symptoms, and this showed potential interstitial lung abnormalities in 58%, which demonstrates the potential for earlier diagnosis in a proportion of patients with IPF. Time to diagnosis did not associate with survival, illustrating that more work needs to be done on prevention of disease progression in patients with pulmonary fibrosis.

## Supplementary Information

**Additional file 1.** **Table S1:** Indications for chest X-rays done prior to the start of symptoms in 96 patients with IPF. **Table S2:** additional information on radiographic findings in 56 patients with potential interstitial lung abnormalities detected on chest X-ray prior to start of symptoms of idiopathic pulmonary fibrosis. Note that the chest X-ray and CT-scan description was based on radiology reports and not on revision of the images. Negative time between chest X-ray and CT-scan indicates that the CT-scan was made before the chest X-ray. **Table S3:** additional information on radiographic findings in 17 patients with potential interstitial lung abnormalities detected on CT-scan prior to start of symptoms of idiopathic pulmonary fibrosis (17 other patients with potential interstitial lung abnormalities on CT-scan that are included in eTable 2 are not included here, so no patients in this table have abnormalities on chest X-ray). Note that the chest X-ray and CT-scan description was based on radiology reports and not on revision of the images. Negative time between CT-scan and chest X-ray indicates that the chest X-ray was made before the CT-scan.

## Data Availability

The datasets generated and/or analysed during the current study are not publicly available due to privacy concerns but are available from the corresponding author on reasonable request.

## Abbreviations

CT: Computed tomography
ILD: Interstitial lung disease
IPF: Idiopathic pulmonary fibrosis
IQR: Interquartile range
UIP: Usual interstitial pneumonia
