# Supplementary material for: Potential interstitial lung abnormalities on chest X-rays prior to symptoms of idiopathic pulmonary fibrosis
Source: BMC Pulm Med. 2022 Aug 30;22:329. doi: 10.1186/s12890-022-02122-8 (PMC9426013; doi:10.1186/s12890-022-02122-8)
Supplement: Supplementary file 1 — Additional file 1. Table S1: Indications for chest X-rays done prior to the start of symptoms in 96 patients with IPF. Table S2: additional information on radiographic findings in 56 patients with potential interstitial lung abnormalities detected on chest X-ray prior to start of symptoms of idiopathic pulmonary fibrosis. Note that the chest X-ray and CT-scan description was based on radiology reports and not on revision of the images. Negative time between chest X-ray and CT-scan indicates that the CT-scan was made before the chest X-ray. Table S3: additional information on radiographic findings in 17 patients with potential interstitial lung abnormalities detected on CT-scan prior to start of symptoms of idiopathic pulmonary fibrosis (17 other patients with potential interstitial lung abnormalities on CT-scan that are included in eTable 2 are not included here, so no patients in this table have abnormalities on chest X-ray). Note that the chest X-ray and CT-scan description was based on radiology reports and not on revision of the images. Negative time between CT-scan and chest X-ray indicates that the chest X-ray was made before the CT-scan. [file 12890_2022_2122_MOESM1_ESM.docx]

**Supplementary Material**

**Potential interstitial lung abnormalities on chest X-rays prior to symptoms of idiopathic pulmonary fibrosis**

T.W. Hoffman^1^, H.W. van Es^2^, D.H. Biesma^3^, and J.C. Grutters^1,4^

1. Interstitial Lung Diseases Center of Excellence, Department of Pulmonology, St. Antonius Hospital, Nieuwegein, The Netherlands

2. Department of Radiology, St. Antonius Hospital, Nieuwegein/Utrecht, The Netherlands

3. Department of Internal Medicine, Leiden University Medical Center, Leiden, The Netherlands

4. Division of Heart and Lungs, University Medical Center, Utrecht, The Netherlands

Corresponding author: T.W. Hoffman, Department of Pulmonology, St. Antonius Hospital, Koekoekslaan 1, 3435 CM, Nieuwegein, The Netherlands; e-mail: [t.hoffman@antoniusziekenhuis.nl](mailto:t.hoffman@antoniusziekenhuis.nl); telephone: 088-3201425

Competing interest: The authors declare that they have no competing interests.

**Table S1:** Indications for chest X-rays done prior to the start of symptoms in 96 patients with IPF

| **Indication** | **N patients** |
| --- | --- |
| Prior to/after thoracic surgery | 12 |
| Cough (temporary) | 10 |
| Chest pain | 6 |
| Dyspnoea (temporary) | 5 |
| Follow-up after pneumonia | 5 |
| Trauma | 3 |
| Crackles | 2 |
| Follow-up for malignancy | 2 |
| Haemoptysis | 1 |
| Family history | 1 |
| Heart murmur | 1 |
| Tuberculosis screening | 1 |
| Tiredness | 1 |
| Recurrent infections | 1 |
| Unknown | 45 |

**Table S2:** additional information on radiographic findings in 56 patients with potential interstitial lung abnormalities detected on chest X-ray prior to start of symptoms of idiopathic pulmonary fibrosis. Note that the chest X-ray and CT-scan description was based on radiology reports and not on revision of the images. Negative time between chest X-ray and CT-scan indicates that the CT-scan was made before the chest X-ray.

| **Patient** | **Time between chest X-ray and start of symptoms (months)** | **Chest X-ray description** | **Time between chest X-ray and CT-scan (months)** | **CT-scan findings** |
| --- | --- | --- | --- | --- |
| 1 | 101 | Increased interstitial markings bilaterally | 12 | Fibrotic changes |
| 2 | 34 | Interstitial abnormalities |  |  |
| 3 | 12 | Fibrotic changes |  |  |
| 4 | 60 | Fibrotic abnormalities |  |  |
| 5 | 24 | Interstitial abnormalities | 1 | Fibrotic changes |
| 6 | 56 | Fibrotic abnormalities | 1 | Fibrotic abnormalities |
| 7 | 51 | Increased subpleural reticulation |  |  |
| 8 | 73 | Increased interstitial markings | 11 | Some fibrotic changes |
| 9 | 12 | Fibrotic changes |  |  |
| 10 | 27 | Increased reticular markings in the right lung |  |  |
| 11 | 45 | Interstitial abnormalities | 1 | Fibrotic abnormalities |
| 12 | 58 | Some interstitial abnormalities |  |  |
| 13 | 120 | Fibrotic abnormalities |  |  |
| 14 | 35 | Interstitial abnormalities |  |  |
| 15 | 33 | Fibrotic abnormalities | 4 | Interstitial lung abnormalities |
| 16 | 44 | Interstitial abnormalities, especially in the righ lung | 1 | Fibrotic abnormalities |
| 17 | 4 | Interstitial abnormalities | -5 | Minimal reticulation |
| 18 | 106 | Fibrotic abnormalities | 75 | Fibrotic abnormalities |
| 19 | 33 | Fibrotic abnormalities |  |  |
| 20 | 104 | Increased interstitial markings in lower and middle fields as well as right upper field |  |  |
| 21 | 64 | Increased interstitial markings |  |  |
| 22 | 92 | Somewhat increased interstitial markings |  |  |
| 23 | 67 | Somewhat increased interstitial markings |  |  |
| 24 | 20 | Interstitial abnormalities bilaterally |  |  |
| 25 | 74 | Interstitial abnormalities, possibly redistribution | 63 | Fibrotic abnormalities |
| 26 | 50 | Reticular abnormalities |  |  |
| 27 | 6 | Interstitial abnormalities, especially in the right lung |  |  |
| 28 | 10 | Interstitial abnormalities |  |  |
| 29 | 7 | Increased markings in both pleural sinuses |  |  |
| 30 | 15 | Interstitial abnormalities | 2 | Fibrotic abnormalities |
| 31 | 15 | Increased interstitial markings, somewhat more on the right |  |  |
| 32 | 37 | Interstitial abnormalities |  |  |
| 33 | 65 | Fibrotic changes | 48 | Fibrotic changes |
| 34 | 34 | Fibrotic changes bilaterally |  |  |
| 35 | 23 | Increased interstitial markings peripherally | 1 | Fibrosis |
| 36 | 3 | Fibrotic changes in the lower zones peripherally |  |  |
| 37 | 70 | Somewhat increased interstitial markings | 2 | Small airways disease |
| 38 | 40 | Interstitial abnormalities |  |  |
| 39 | 86 | Fibrotic abnormalities |  |  |
| 40 | 73 | Increased interstitial markings | 20 | Subpleural fibrotic changes |
| 41 | 212 | Bilateral reticular and nodular abnormalities | 1 | No abnormalities |
| 42 | 24 | Increased interstitial markings |  |  |
| 43 | 12 | Increased interstitial markings bilaterally in the lower fields |  |  |
| 44 | 24 | Interstitial abnormalities |  |  |
| 45 | 126 | Fibrotic abnormalities |  |  |
| 46 | 78 | Bronchopathic and fibrotic abnormalities |  |  |
| 47 | 71 | Somewhat increased interstitial markings |  |  |
| 48 | 91 | Fibrotic abnormalities | 3 | Fibrotic changes |
| 49 | 26 | Increased interstitial markings especially peripherally | 1 | Bilateral subpleural reticulation |
| 50 | 73 | Increased interstitial markings |  |  |
| 51 | 30 | Increased interstitial markings, especially in the left lower zone |  |  |
| 52 | 101 | Some fine changes, especially in the left lower zone |  |  |
| 53 | 51 | Fibrotic abnormalities | 1 | Fibrotic changes |
| 54 | 53 | Reticular abnormalities |  |  |
| 55 | 43 | Interstitial abnormalities |  |  |
| 56 | 43 | Diffusely increased interstitial markings |  |  |

**Table S3:** additional information on radiographic findings in 17 patients with potential interstitial lung abnormalities detected on CT-scan prior to start of symptoms of idiopathic pulmonary fibrosis (17 other patients with potential interstitial lung abnormalities on CT-scan that are included in eTable 2 are not included here, so no patients in this table have abnormalities on chest X-ray). Note that the chest X-ray and CT-scan description was based on radiology reports and not on revision of the images. Negative time between CT-scan and chest X-ray indicates that the chest X-ray was made before the CT-scan.

| **Patient** | **Time between CT-scan and start of symptoms** | **CT-scan findings** | **Time between CT-scan and chest X-ray** | **Potential interstitial lung abnormalities on chest X-ray** |
| --- | --- | --- | --- | --- |
| 1 | 40 | Some fibrotic abnormalities |  |  |
| 2 | 82 | Some fibrotic abnormalities | -2 | No |
| 3 | 44 | Mild fibrosis |  |  |
| 4 | 52 | Normal |  |  |
| 5 | 9 | Mild fibrosis |  |  |
| 6 | 43 | Mild fibrotic abnormalities in lower fields (abdominal scan) |  |  |
| 7 | 43 | Some fibrosis |  |  |
| 8 | 108 | Normal |  |  |
| 9 | 60 | Normal |  |  |
| 10 | 62 | Normal | 1 | No |
| 11 | 142 | Some bronchiectasis and alveolar infiltrates | 12 | No |
| 12 | 29 | Mild fibrosis |  |  |
| 13 | 68 | Normal | 0 | No |
| 14 | 110 | Some fibrotic abnormalities | -30 | No |
| 15 | 33 | Normal | 28 | No |
| 16 | 18 | Fibrotic abnormalities in lower fields  (abdominal scan) |  |  |
| 17 | 48 | Normal | -240 | No |
| 18 | 34 | Subtle interstitial lung abnormalities | 1 | No |
| 19 | 99 | Some fibrotic abnormalities in right upper lobe |  |  |
| 20 | 40 | Fibrotic abnormalities | -108 | No |
| 21 | 84 | Minimal fibrotic changes | 2 | No |
| 22 | 84 | Normal | 47 | No |
| 23 | 46 | Subpleural reticulation |  |  |
| 24 | 54 | Bilateral fibrotic abnormalities |  |  |
| 25 | 42 | Some fibrosis |  |  |
